# Mineral Speciation for CO_2_ Captured by Potassium Hydroxide

**DOI:** 10.1002/open.202500376

**Published:** 2025-10-14

**Authors:** Ehsan Ezzatpour Ghadim, Stephanie Bachmann, Rodrigo S. Correa, Dinu Iuga, Joanna F. Collingwood, Peter J. Sadler

**Affiliations:** ^1^ School of Engineering University of Warwick Coventry CV4 7AL UK; ^2^ Department of Physics University of Warwick Coventry CV4 7AL UK; ^3^ Department of Chemistry University of Warwick Gibbet Hill Road Coventry CV4 7AL UK; ^4^ Department of Chemistry Federal University of Ouro Preto Ouro Preto 35402‐136 Minas Gerais Brazil

**Keywords:** High field ^1^H, ^13^C, and ^39^K NMR, Carbon dioxide capture, Potassium bicarbonate and carbonate, Single‐crystal X‐ray diffraction, Powder X‐ray diffraction

## Abstract

Capture of greenhouse gases, especially CO_2_, can reduce the effects of global warming and generate valuable minerals as feedstock for industry. Herein, the mineral products formed by capture of atmospheric CO_2_ by potassium hydroxide (KOH) in aqueous, aqueous‐ethanol, and aqueous‐acetone solutions, and aqueous‐acetone enriched using solid CO_2_ are studied. A multimodal analysis combining single‐crystal X‐ray diffraction (SCXRD), powder X‐ray diffraction (PXRD), with Pawley and Rietveld refinements, and 850 MHz, 1 GHz, and 1.2 GHz ^1^H, as well as ^13^C, and ^39^K nuclear magnetic resonance (NMR), is used to analyze the composition of the mineral products. SCXRD identifies KHCO_3_ in space group *P*2_1_/*n* (transformable to *P*2_1_/*a*) as a product from all reactions. PXRD and NMR data show the presence of both crystalline and amorphous phases in products, predominantly as mixtures of KHCO_3_ and K_2_CO_3_ and its hydrates, with KOH as a minor component, except for aqueous‐ethanol which gives KHCO_3_ in high purity. Analysis of complex ^1^H NMR data is aided by 2D nuclear Overhauser effect spectroscopy (1 GHz), which characterizes C—OH···O=C interactions. Revealing K_2_CO_3_ hydration is aided by deconvolution of ultrahigh‐field 28.2 T (56 MHz) ^39^K spectra. This multimodal approach provides new insights into the speciation of potassium minerals from CO_2_ capture.

## Graphical abstract

Multimodal and deconvolution analysis of the crystalline and amorphous mineral products from the rapid capture of CO_2_ in aqueous, aqueous‐ethanol, and aqueous‐acetone solutions of potassium hydroxide by complementary single‐crystal and powder X‐ray diffraction, and high field ^1^H, ^13^C, and ^39^K NMR, provides new insight into the speciation of both KHCO_3_‐ and K_2_CO_3_‐rich crystalline and amorphous products, with enhanced ^39^K resolution of components at 28 T (56 MHz, 1.2 GHz for ^1^H).

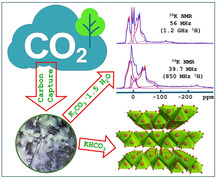

## 1 Introduction

The excessive use of fossil fuels has significantly increased the levels of greenhouse gases, mainly atmospheric CO_2_, which affect climate change, ecosystems, and human livelihoods.^[^ ^1^ ^]^ Carbon capture utilization and storage technologies have emerged to mitigate these effects by capturing atmospheric CO_2_.^[^ ^2^ ^]^ A promising procedure is the capture of carbon through the formation of carbonate minerals. In particular, there is interest in the integrated capture CO_2_ and mineralization processes on a commercial scale.^[^ ^3^ ^]^ Both potassium bicarbonate (KHCO_3_) and potassium carbonate (K_2_CO_3_) have large‐scale usage in a wide variety of applications. These include food (source of CO_2_ for leavening in baking), drinks, medicines, fire extinguishers, agriculture, and fungicides for KHCO_3_,^[^ ^4^ ^]^ and dietary supplements, drying agents, cooking, buffering agents, and animal feeds for K_2_CO_3_.^[^ ^5^ ^]^ Additional interest in the structures of potassium minerals arises from their potential use as solid‐state electrolytes in next‐generation batteries.^[^ ^6^ ^]^

The generation of carbonates from CO_2_ requires hydration of CO_2_, which is a very slow process (taking seconds).^[^ ^7^ ^]^ In humans, this process enables the rapid uptake of CO_2_ in tissues and its release from the lungs. The rate of this aquation/deaquation is catalyzed by the zinc enzyme carbonic anhydrase by a factor of ≈10^7^.^[^ ^8^ ^]^ Zn(II) activates H_2_O by stabilizing the formation of hydroxide, bound as Zn–OH for attack on CO_2_. The enzyme can be used in large‐scale CO_2_ capture processes.^[^ ^9^ ^]^ For photosynthesis in plants, zinc carbonic anhydrase ensures the supply of CO_2_ from bicarbonate near the active site of the CO_2_‐fixation enzyme RuBisCo in chloroplasts.^[^ ^10^ ^]^

In water, hydrated CO_2_, carbonic acid (H_2_CO_3_), has p*K*_a1_ and p*K*_a2_ values of ca. 6.4 and 10.3, corresponding to the formation of bicarbonate (HCO_3_^−^) in the first step, and carbonate (CO_3_^2−^) in the second step. Thus, in near‐neutral pH (7) solutions, the major form will be bicarbonate and in highly alkaline solutions (>pH 11) largely carbonate. It is notable that p*K*_a2_ increases to ≈11.1 in 25% EtOH solutions.^[^ ^11^ ^]^ In a laboratory room, the concentration of CO_2_ in the atmosphere is likely to be ≈500 ppm,^[^ ^12^ ^]^ giving rise to aqueous solution concentrations of ≈30 mM. In the present work, solid CO_2_ (dry ice/cardice) was added to saturate a solution with CO_2_.

This study is focused on comparison of the composition and structures of the potassium (bi)carbonate minerals formed by the capture of CO_2_ in KOH in various chemical conditions. We use a combination of three techniques. First, single‐crystal X‐ray diffraction (SCXRD) which can determine mineral structures at the atomic level, but only for chosen single crystals, which may or may not be representative of the whole sample. Second, powder X‐ray diffraction (PXRD) which can characterize mixtures of crystals in polycrystalline samples, if the observed diffraction pattern can be fitted to a model of the constituent crystal structures. This can be tacked by either a Pawley refinement which fits peak positions, intensities, linewidths to known unit cells, space groups, and instrumental parameters, but does not use atomic coordinates,^[^ ^13^ ^]^ or the Rietveld method which refines a complete structural model, including atomic positions, occupancies, and thermal parameters by fitting the whole PXRD pattern.^[^ ^14^ ^]^ Third, we use multinuclear solid‐state nuclear magnetic resonance (NMR) spectroscopy, in this case observing ^1^H, ^13^C, and ^39^K resonances, which provides structural information on both crystalline and amorphous components of a mineral mixture, via NMR chemical shifts, and in the case of the quadrupolar nucleus ^39^K from its quadrupolar couplings.

Quadrupolar nuclides (I > 1/2) account for nearly 75% of the stable magnetic nuclides in the periodic table, but are difficult to study by NMR.^[^ ^15^ ^]^ The interaction of a nuclear quadrupole moment with electric field gradients arising from the local environment provides a strong relaxation mechanism for spin states often resulting in short‐lived states with broad resonances. Hence, ^39^K resonances are usually broad, unless they are in highly symmetric environments, which reduce the magnitude of the electric field gradients at the nuclei. In recent years, the availability of increasingly high magnetic fields (linearly proportional to resonance frequencies of nuclei) has greatly improved the sensitivity for detection of quadrupolar isotopes and resolution of their peaks in spectra, especially for half‐integer quadrupolar nuclei such as ^39^K (*I* = 3/2) due to the reduction of second‐order quadrupolar broadening and sharpening of the central transition.^[^ ^16^ ^,^ ^17^ ^]^ This is well illustrated here by comparison of ^39^K NMR spectra at 20 and 28 T.

Combining solid‐state NMR spectroscopy, density functional theory (DFT) NMR calculations, and machine learning to consider the dynamics in the systems, Rhodes et al.^[^ ^18^ ^]^ recently used 1D ^17^O (*I* = 5/2, 0.04% natural abundance) solid‐state NMR to study and model the dynamics of CO_2_ capture by hydroxide‐based materials. They compared calculations and experimental data for the model phases K_2_CO_3_·1.5H_2_O and KHCO_3_ as well as for the carbon capturing metal‐organic framework materials. Furthermore, Peach et al.^[^ ^19^ ^]^ used 1D and 2D ^17^O solid‐state NMR and complementary techniques to investigate sodium and potassium (bi)carbonate salts used for carbon capture by enrichment of ^17^O in the salts KHCO_3_, K_2_CO_3_·1.5 H_2_O, NaHCO_3_, Na_2_CO_3_, and Na_2_CO_3_ · H_2_O. ^17^O enrichment of the samples made it possible to investigate them under various conditions including different magnetic field strengths and sample temperatures, e.g., ambient temperature. To gain deeper insights into how the spectra are influenced by the dynamics, subsequent work by Peach et al.^[^ ^20^ ^]^ used enriched samples. They investigated these at low temperatures (e.g., 100 K) at 14.1 and 18.8 T and by complementary gauge including projected augmented wave (GIPAW)‐DFT calculations for the static model as well as by Molecular Dynamics GIPAW‐DFT computations, to consider the dynamics within the system. These calculations revealed that even at 100 K, the local environment, and therefore the ^17^O NMR parameters, are influenced by the dynamics.

Here, the products formed by reactions of atmospheric CO_2_ with KOH in aqueous, aqueous‐acetone, aqueous‐ethanol, and aqueous‐acetone with added dry ice are analyzed using a new trimodal approach which provides insights into the speciation of both crystalline and amorphous mineral products from CO_2_ capture by KOH.

## 2 Results and Discussion

Many previously reported studies of CO_2_ capture have focused on absorption by K_2_CO_3_, as noted by Liu et al.^[^ ^21^ ^]^ Here, mineral samples were prepared by CO_2_ capture from the air in KOH solution on a small scale by mixing equal volumes of KOH (15 mL, 10 M) and H_2_O, and heating to 353 K, followed by crystallization at ambient temperature to give sample **3**, or with initial addition also of EtOH (sample **1**), or acetone (with heating to 333 K, sample **2**) or acetone with further addition of solid CO_2_ (dry ice, sample **4**), as detailed in SI section S1.2.

First, we determined the structures of representative single crystals from each of the four samples by XRD. For bulk analyses of all microcrystals in the samples, we determined their PXRD patterns, and finally recorded their ^1^H, ^13^C, and ^39^K solid‐state NMR spectra as these contain resonances for both amorphous and crystalline forms of the minerals. The presence of H‐bonds was detected by solid‐state ^1^H NMR, whereas their reliable identification by XRD is challenging. Additionally, the deconvolution of ^39^K NMR spectra revealed different components/phases in mixtures of products, especially with the aid of 56 MHz ^39^K NMR (1.2 GHz for ^1^H).

## 3 SCXRD

**Figure 1:**
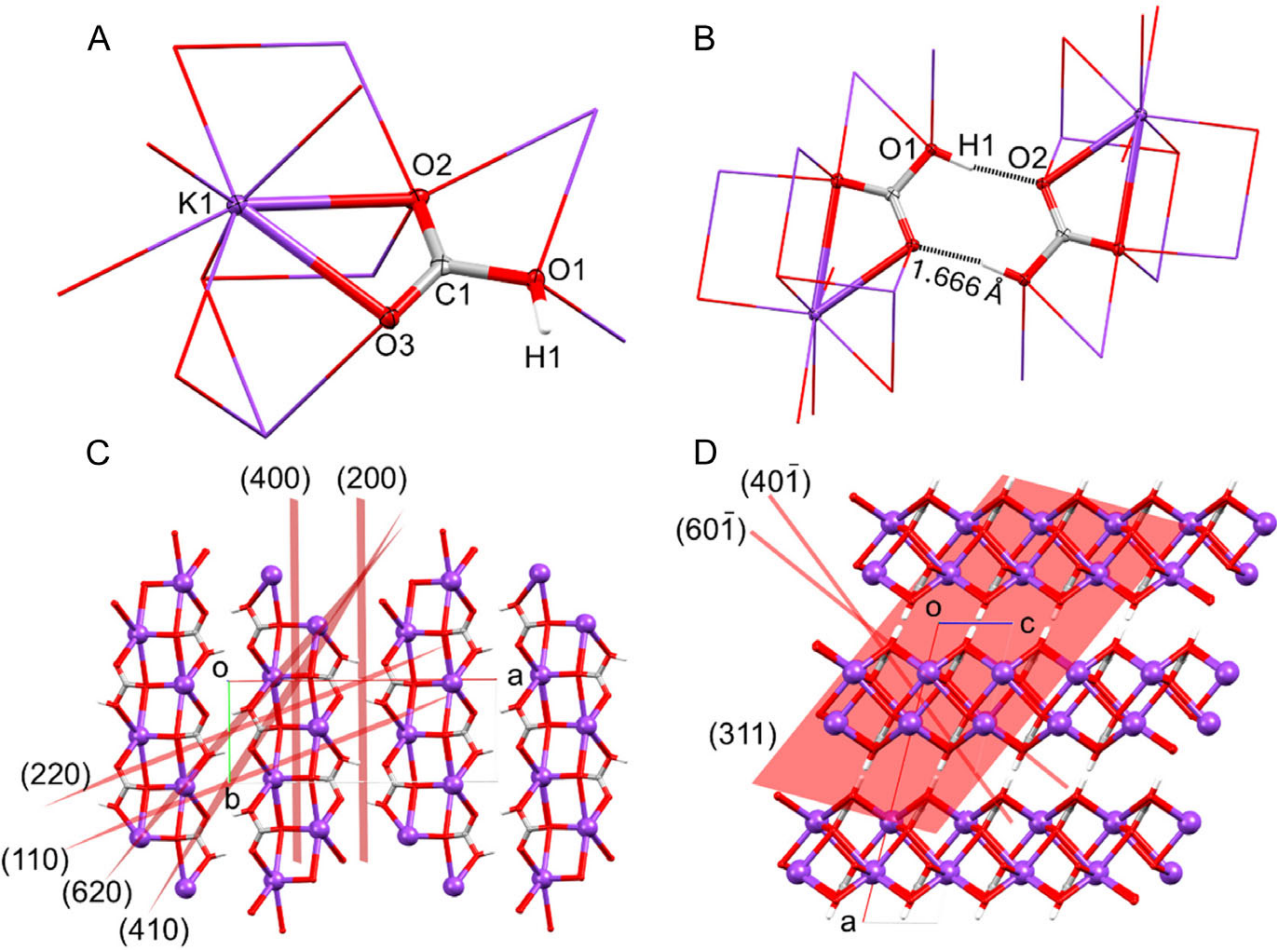
Major features of the X‐ray crystal structure of KHCO_3_ isolated from samples **1**–**4**. A) Asymmetric unit of KHCO_3_ with atom labels and thermal ellipsoids shown at 50% of probability and symmetry‐equivalent atoms surrounding K and O depicted in wireframe mode. B) Centrosymmetric hydrogen bonds, highlighting short O1—H1···O2 (1.666 Å) contacts. Characteristic peaks related to these features are seen in the NMR spectra (see Figure 3B). C) Packing view along the *c‐*axis and D) view along the *b*‐axis. Both images show parallel layers formed by (KHCO_3_)_*n*_ chains linked via K—O bonds. These layers are connected to each other by hydrogen bonds. Selected Miller planes (*hkl*) corresponding to the peaks observed in the PXRD pattern at various 2*θ* values (see Figure 2) are shown: plane (200) peak at 12°; (110) 17°; (400) 24°; (410) 29°, (40‐1) 30.5°; (220) 34.2°; (311) 38.2°; (60‐1) 39°; (620) 49.5°.

For SCXRD, at least four crystals from each of samples **1**–**4** were screened to ensure that the crystal chosen for data collection was representative. The X‐ray crystallographic information is in Supporting Information section S2.1, Tables S1 and S2. The crystals from samples **1**–**4** each had the same composition of KHCO_3_ (**Figure** **1**) The crystal structures were initially solved as KHCO_3_ in a monoclinic system (*P*2_1_/*n* space group) with the unit cell parameters [*a* = 3.6587(2), *b* = 5.6021(3), *c* = 14.6647(6) Å, *β* = 90.032(4)°]. This unit cell had not previously been reported and initially raised the possibility that it might represent a new polymorph. However, subsequent analysis showed that this structure is identical to those previously reported with *P*2_1_/*a* space group [*a* = 15.115(2), *b* = 5.6044(10), *c* = 3.6609(10) Å, *β* = 103.984(20)°], in which the *P*2_1_/*n* space group can be transformed to *P*2_1_/*a* by a matrix transformation (see S1.2).^[^ ^22^ ^]^ The unit cell parameters for samples **1**–**4** indicate the presence of the same crystalline phase. Notably, high‐quality crystallographic data were obtained with *R* factors of ≈2 (Table S1, Supporting Information).

Our redeterminations confirm that the asymmetric unit of KHCO_3_ consists of one potassium, three oxygen, and one hydrogen atom (Figure 1). The K^+^ ion is 8‐coordinate with K–O distances ranging from 2.68 to 3.01 Å, (Table S2, Supporting Information). The K–K distances are all ≈3.65 Å. The crystal packing consists of parallel layers of KHCO_3_ separated by 2.329 Å. These layers are connected by strong centrosymmetric intermolecular hydrogen bonds with O1—H1···O2 contacts of 1.666 Å and an O1···O2 separation of 2.590 Å (Figure 1B) which stabilizes the crystal packing (Figure 1C,D). These data were collected at 100 K where the H atoms are well ordered, whereas it is reported that at > 318 K, the H atoms become disordered, resulting in a phase transition from *P*2_1_/*a* to *C*2/*m* space group.^[^ ^23^ ^]^

## 4 PXRD Analysis of Mineral Products

PXRD analysis was carried out to investigate whether the single crystals of potassium bicarbonate, KHCO_3_, isolated from samples **1** to **4** are representative of the bulk of the mineral products from CO_2_ capture. Rietveld refinement fits a complete crystallographic model to the PXRD pattern by least squares, refining lattice parameters, atomic positions, phase fractions, peak shapes, microstructure, and instrumental effects simultaneously.^[^ ^14^ ^]^ In contrast, Pawley refinement refines only individual intensities without a structural model.^[^ ^13^ ^]^ Rietveld's method uses physical scattering factors and symmetry to resolve overlapping peaks, corrected background, and preferred orientation, and delivers robust quantitative and structural results for complex, multiphase samples.^[^ ^14^ ^,^ ^24^ ^]^ By leveraging the full diffraction pattern rather than single peaks, Rietveld refinement has become the standard for precise phase quantification, microstructural analysis, and detection of subtle structural distortions in inorganic, organic, and hybrid materials.^[^ ^25^ ^]^

**Figure 2:**
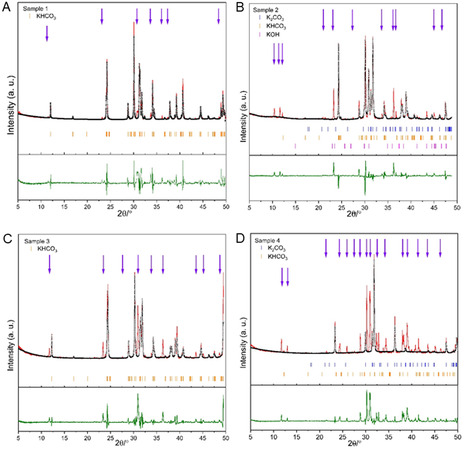
PXRD diffraction patterns for various known, and unidentified, phases (purple arrows), assigned. A–D) For samples **1**–**4**, respectively, show the experimental pattern (black points), differences (green), Pawley fit (red line), and allowed Bragg peak positions for K_2_CO_3_ in blue, KHCO_3_ in orange, and KOH in pink. The variations from predicted signal intensities can be ascribed to the partial alignment of the microcrystals in the powder used for the PXRD measurements.

Analysis of PXRD data for samples **1**–**4** confirmed the presence of both K_2_CO_3_ and KHCO_3_ phases, see **Figure** **2**. Sample **1** (KOH/water/ethanol) and sample **3** (KOH/water) show patterns closely aligned with KHCO_3_. In contrast, samples **2** (KOH/water/acetone) and **4** (KOH/water/acetone/dry ice) show higher similarity also to potassium carbonate, K_2_CO_3_. Notably, acetone is likely to increase the rate of solvent evaporation and crystal growth compared to ethanol or water. In addition, sample **4** (prepared with dry ice present) matches more closely to the simulated pattern of K_2_CO_3_, suggesting that regulating the CO_2_ concentration plays a role in reversing the phases between K_2_CO_3_ and KHCO_3_. The dry ice is likely to increase the solution concentration of CO_2_ from ≈33 mM to ≈1 M or higher.^[^ ^26^ ^]^

PXRD patterns of KHCO_3_, K_2_CO_3_, KOH, and their hydrates are notoriously difficult to refine because their structures are closely related, and their strongest reflections frequently coincide.^[^ ^27^ ^]^ Pure KOH can react with CO_2_ within minutes in the air, so traces of KHCO_3_ or K_2_CO_3_·1.5H_2_O are almost inevitable in laboratory samples.^[^ ^14^ ^]^ The Pawley refinement for sample **1** shows a good match with KHCO_3_ (Table S3, Supporting Information)_,_ confirming an almost single‐phase potassium bicarbonate. In sample **2**, KHCO_3_ remains dominant; however, additional peaks for K_2_CO_3_ coincide with those for a small amount of KOH. Sample **3** shows a major KHCO_3_ phase; however, some unidentified extra peaks are also present (purple arrows in Figure 2). The major component in sample **4** is K_2_CO_3_, with a minor KHCO_3_ contribution and a diffuse background that suggests poorly ordered or hydrated material. It is surprising that the KOH/water/acetone/dry ice solution does not result in KHCO_3_ as the major component of sample **4** since it is more acidic, perhaps a consequence of kinetics and relative lattice energies dominating product formation, including mixed minerals. Further investigation of the mechanisms of product formation was beyond the scope of the present work.

Interpretation of the 30–33° 2*θ* (Cu Kα) region is difficult because the KHCO_3_ (211), K_2_CO_3_ (220), and KOH (101) peaks overlap. Major K_2_CO_3_ reflections at 30.1°, 32.2°, and 34.3° further reinforce the congestion, and additional overlaps at 18–19°, 29–30°, and 34–35° include intense bicarbonate reflections because Pawley refinement distributes the total intensity among coincident lines.^[^ ^13^ ^]^ Quantitative Rietveld refinement of complex carbonate mixtures demonstrates that excluding even minor phases can have a significant impact on the least‐squares algorithm, causing it to apportion intensity to the remaining models and resulting in biased phase fractions and highly correlated parameters. Rietveld analysis resolves peak overlap only if every crystalline constituent (anhydrous K_2_CO_3_, KHCO_3_, KOH, and hydrates) is entered with an accurate structural model from the ICSD databases, **Table** **1**.

**Table 1:** Phase weight fractions (wt%) and goodness‐of‐fit (GOF) values from Rietveld refinement of PXRD data for samples **1–4**. Diffraction pattern simulations are based on single‐crystal structures from the ICSD: KHCO_3_ (2327), K_2_CO_3_ (662), and KOH (47114).

Samples	KHCO_3_ [%]	K_2_CO_3_ [%]	KOH [%]	GOF
**1**	100	–	–	4.65
**2**	58.4	24.7	16.9	5.76
**3**	100	–	–	6.2
**4**	37.07	62.30	–	5.51

Therefore, reasonable assignments from the quantitative multiphase Rietveld refinement are sample **1**, KHCO_3_, sample **2**, mainly KHCO_3_ with smaller amounts of K_2_CO_3_ or its sesquicarbonate and KOH, sample **3**, KHCO_3_ with minor additional species, and sample **4**, mainly K_2_CO_3_ and a smaller KHCO_3_ fraction, as well as disordered material.

## 5 Solid‐State ^1^H, ^13^C, and ^39^K NMR

Solid‐state NMR is a powerful technique for elucidating the atomic structure of potassium (bi)carbonate minerals, complementing the data obtained by SCXRD as well as PXRD. Here, we use a combination of ^1^H (natural abundance 99.9855%, nuclear spin quantum number *I* = ½), ^13^C (1.06%, *I* = ½), and ^39^K NMR (natural abundance 93.3%, *I* = 3/2). For ^39^K, Moudrakovski et al.^[^ ^28^ ^]^ (and refs therein) have shown that at very high magnetic field (21 T, ^1^H 900 MHz), the effects of quadrupolar interactions are reduced significantly and the sensitivity and accuracy in determining chemical shifts and quadrupolar coupling parameters improve dramatically. In addition, inequivalent but similar potassium sites in crystals can show chemical shift differences of more than 10 ppm.

Based on the PXRD data, it can be assumed that samples **1‐** **4** are potentially mixtures of several amorphous or crystalline phases consisting of the NMR active nuclei, ^1^H, ^13^C, ^17^O (abundance 0.038%), and ^39^K. To assign the NMR signals, we selected a set of eight different crystal structures to predict the chemical shifts as well as the quadrupolar coupling constant (*C*_Q_) for ^39^K.

**Figure 3:**
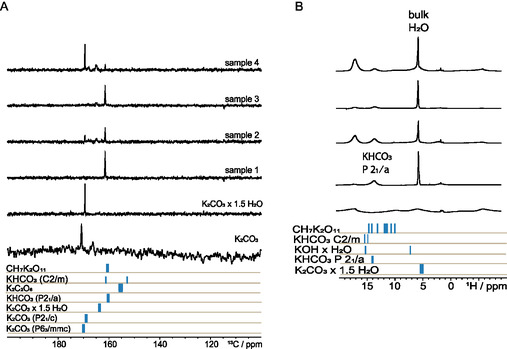
Solid‐state NMR spectra of samples **1**–**4**. A) 214 MHz ^13^C CPMAS spectra recorded at a spinning frequency of 10 kHz, 64 scans, contact time of 1 ms, and an interscan delay of 3 s for K_2_CO_3_·1.5H_2_O and 10 s for the other samples. For K_2_CO_3_ the spectrum was acquired at 251.5 MHz for ^13^C using single pulse excitation, 28 scans with 100 s delay. B) 850 MHz ^1^H solid‐state NMR spectra recorded at a spinning frequency of 10 kHz, 8 scans, and an interscan delay of 5 s. Below the experimental spectra are the chemical shifts based on CASTEP/GIPAW calculations.^[^ ^40^, ^41^ ^–^ ^42^ ^]^

**Figure** **3** shows the ^13^C cross‐polarization magic angle spinning (CPMAS) spectra of samples **1**–**4** as well as the CPMAS spectrum of K_2_CO_3_·1.5H_2_O. The ^13^C signal of K_2_CO_3_ at 171 ppm (calculated: 170.9 ppm) is very close to the ^13^C signal of K_2_CO_3_·1.5H_2_O at 169.5 ppm (calculated: 164 ppm), making it very difficult to identify the hydration state of K_2_CO_3_ (**Table** **2**). This signal can be assigned to the CO_3_^2−^ unit of the crystal structure. The ^13^C signals for K_2_CO_3_ and its sesquihydrate are reported to be in a similar region: 171.2^[^ ^29^ ^]^ and 170.7^[^ ^30^ ^]^ ppm for K_2_CO_3_ and 169.8^[^ ^19^ ^]^ and 169.9 ppm for K_2_CO_3_·1.5H_2_O. K_2_CO_3_ and its sesquihydrate are highly hygroscopic materials.^[^ ^31^ ^]^ The latter has the only reported X‐ray crystal structure for hydrated K_2_CO_3_.

The ^13^C spectra of the samples show that the signal at 169.5 ppm is present in samples **2** and **4**, where a clear ^1^H signal assigned to bulk water can be observed; it can be therefore assumed that the present structure is the sesquihydrate of K_2_CO_3_. This is a minor component in sample **2**, whereas it is the major species in sample **4** and not present in the spectra of samples **1** and **3** within the limit of detection. The ^13^C signal at 161.6 ppm can be assigned to the carbon environment of KHCO_3_ which is present in all samples.^[^ ^25^ ^]^ For KHCO_3,_ two crystal structures are known, with the space group *P2*_1_/*a* present until the sample is heated to 318 K, at which point it transforms to its high‐temperature crystal form with the space group *C*2/*m*.^[^ ^32^ ^,^ ^33^ ^]^ Based on this investigation, the space group *P2*_1_/*a* was used for further evaluation. Furthermore, ^13^C spectra of both **2** and **4** show several new chemical environments between the two main signals. These signals are less distinct and broad and are assignable to less ordered environments; however, these signals are not assignable in the PXRD patterns. They appear in an area where ^13^C signals of K_2_CO_3_·1.5H_2_O or K_2_CO_3_ (*P*6_3_/*mmc*) can be expected based on calculations (Table S4, Supporting Information). However, clear signal assignments are not possible as all crystal structures are similar, and the calculation of the chemical shifts is not precise enough. The calculated ^13^C shifts of dipotassium peroxo carbon dioxo 3.5‐peroxide (K_2_(O_2_)CO_2_(H_2_O_2_)_3.5_) and K_2_C_2_O_6_ are below *δ*(^13^C) = 160 ppm where no signals in the spectrum can be observed and therefore can be excluded for these samples. For all ^13^C CPMAS and direct polarization magic angle spinning (DPMAS) spectra, no peaks for residual organic solvent (ethanol or acetone) were observed (Figure S1, Supporting Information).

Nonetheless, the ^1^H NMR spectra of the four samples, as well as of K_2_CO_3_·1.5H_2_O (Figure 3B), show that water was present in all samples. The main water signal at *δ*(^1^H) = 5.1 ppm can be assigned to clustered water^[^ ^27^ ^,^ ^34^ ^]^ and to the hydrated form of K_2_CO_3_.

The two ^1^H NMR signals between *δ*(^1^H) = 13 and 18 ppm (Figure 3B) are broad and therefore likely to be OH peaks. In this region, peaks such as COOH can also be observed. Combined with the information from the ^13^C spectra and the calculations, it can be assumed that the signal at *δ*(^1^H) = 13 ppm can be assigned to KHCO_3_ and its H bonds (Figure 1B). Samples **2** and **4** as well as K_2_CO_3_·1.5H_2_O show an intense and broad signal at *δ*(^1^H) = 17 ppm. From the shape, this signal belongs to an OH group and is likely due to a strong proton donor, given the large shift to low field. The K_2_CO_3_·1.5H_2_O had been dried prior to the experiments, which explains why the signal at 5.1 ppm is broad since only strongly bound surface water remained. The 1 GHz ^1^H–^1^H NOESY data with a mixing time of 800 ms (Figure S2, Supporting Information) for sample **2** (recorded ≈ 3 weeks later) show that the proton with a resonance at ≈17 ppm is in spatial proximity to that with a signal at ≈7 ppm.

**Table 2:** Assignment of ^1^H and ^13^C NMR chemical shifts of samples **1**–**4** compared to values from the literature.

Sample	^1^H (ppm) assignment			^13^C (ppm) assignment			
	–OH	–OH	Bulk H_2_O	CO_3_^2−^(171 ± 1)^[^ ^19^, ^29^, ^30^, ^31^ ^]^			HCO_3_^−^(161 ± 1)^[^ ^25^ ^]^
**1**	17.7, 16.8, 16.4, 15.6	13.0	5.0	–	–	–	161.6
**2**	16.8, 16.5	13.0	5.1	169.5	168.0, 167.2	164.8	161.5
**3**	16.9, 16.5	13.0	5.1	–	168.4	165.1	161.6
**4**	16.9, 16.7, 16.5	13.0	5.1	169.5	168.0	165.1	161.5
K_2_CO_3_·1.5H_2_O	17	–	5.1	169.5	–	–	–
K_2_CO_3_				171.0			

**Figure 4:**
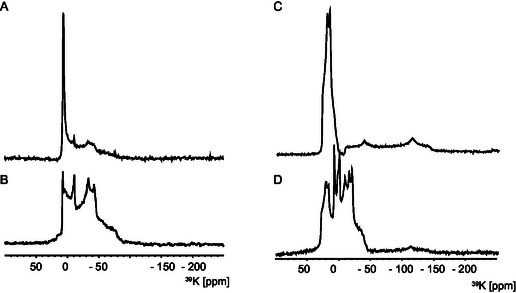
39.7 MHz ^39^K spectra of A) K_2_CO_3_·1.5H_2_O hydrate B) K_2_CO_3_·1.5H_2_O dried for 15 min at 353 K and stored in vacuum for an additional 15 min. (B). Spectra were acquired with 1408 and 15,088 scans, respectively, an interscan delay of 2 s and a double frequency sweep (dfs) echo sequence.^[^ ^43^ ^]^ C) 56 MHz ^39^K spectra of anhydrous K_2_CO_3_ (K_2_CO_3_·1.5H_2_O was dried for 15 min at 448 K and stored in vacuum for additional 15 min). D) Anhydrous K_2_CO_3_ were exposed to atmospheric humidity for 15 min (56 MHz spectrum is in Figure S7, Supporting Information). Spectra were acquired with 51,824 and 22,536 scans, respectively, an interscan delay of 1 s, with single‐pulse excitation.

The effect of hydration on K_2_CO_3_ was strongly revealed by ^39^K NMR. **Figure** **4**A shows the ^39^K spectrum of hydrated K_2_CO_3_. Drying the hydrated sample for 15 min at 353 K and storing in vacuum for an additional 15 min removed most of the superficial water and two ^39^K sites of K_2_CO_3_·1.5H_2_O can be identified (Figure 4B, Figure S6B, Supporting Information). Further drying of the sample at 448 K for 15 min and storing in vacuum for an additional 15 min produced anhydrous K_2_CO_3_ (Figure 4C, Figure S6A, Supporting Information). However, the rehydration process had already begun after exposing the sample to atmospheric humidity for 10 min, Figure 4D. The deconvolution of the spectra, shown in Figure 4B, is in Figure 6, and deconvolution of the spectra, shown in Figure 4C,D, is in Figures S4 and S5, Supporting Information.

**Figure 5:**
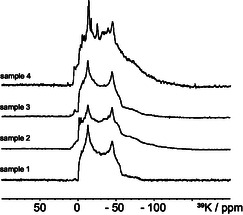
39.7 MHz ^39^K spectra of samples **1–4.** The spectra were acquired with 1480, 1480, 27,336, and 4096 scans, respectively; an interscan delay of 2 s and a dfs echo sequence^[^ ^43^ ^]^ were used to acquire the spectra. The chemical shift reference was the ^39^K signal of KBr, *δ*(^39^K) = 55.4 ppm.^[^ ^28^ ^]^

The ^39^K MAS spectra of all four samples are shown in **Figure** **5**. The ^39^K spectra of samples **1**, **2**, and **3** show mostly a single ^39^K site with quadupolar parameters similar to those for KHCO_3_. For sample **4**, the ^39^K spectrum (Figure 5 top) looks more complex, but can be deconvoluted (see Figure SI6, Supporting Information) as both KHCO_3_ and K_2_CO_3_·1.5H_2_O. Due to the small amount of species other than KHCO_3_ and K_2_CO_3_·1.5H_2_O, it is likely that these species cannot be observed in the ^39^K spectra as distinct signals. The main components of the samples are KHCO_3_ and K_2_CO_3_·1.5H_2_O.

**Figure 6:**
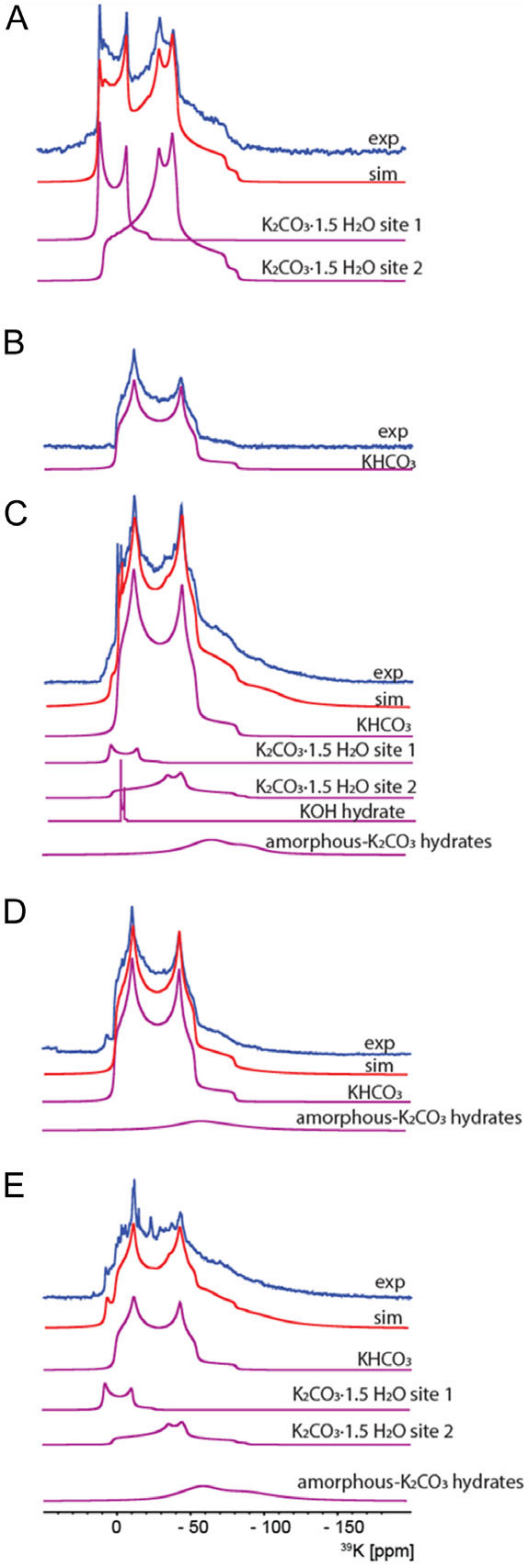
39.7 MHz ^39^K spectra of A) K_2_CO_3_·1.5H_2_O and B–E) samples **1**–**4**, respectively. The experimental spectra are shown in blue superimposed with the sum of sites considered for simulation in red. K_2_CO_3_·1.5H_2_O is simulated with 2 sites with the values in Table 3. The spectra of samples **1** and **3** are simulated as a single component with C_Q_ and *η*_Q_ similar to the literature values for KHCO_3_,^[^ ^28^ ^]^ and **2** and **4** using both the two K_2_CO_3_·1.5H_2_O sites and the single site of KHCO_3_ (purple).^[^ ^28^ ^]^

In anhydrous K_2_CO_3_, the two K^+^ sites (an axially symmetric site‐1, hexacoordinated with a *C*_Q_ = 1 MHz and a site‐2, octacoordinated with *C*_Q_ = 3.3 MHz, see Figure S6A, Supporting Information) are in a ratio of 1:1 (experimental ^39^K spectral deconvolution ratio shown in Figures S4, Supporting Information, is 56% site‐1 and 44% site‐2). Upon hydration, water binds to both K^+^ sites resulting in three distinct sites according to Skakle et al.^[^ ^35^ ^]^ Figure S6B, Supporting Information, shows that the coordination numbers in the three K^+^ sites 1, 2, and 3 are 8, 10, and 7, respectively, all with O‐ligands, or four distinct sites according to Rhodes et al.^[^ ^18^ ^]^ (see Table S4, Supporting Information). Deconvolution of the ^39^K MAS spectrum of K_2_CO_3_·1.5H_2_O shown in **Figure** **6**A was carried out by considering two sites (parameters given in **Table** **3**) in a ratio of 24% for site 1 (with axial symmetry) and 76% for site 2.

**Table 3:** ^39^K NMR parameters determined in this work and those reported in the literature.

Standards	Parametera)						
	Site	*δ*_iso_ this work [ppm]	*δ*_iso_^[^ ^28^ ^,^ ^36^ ^]^ [ppm]	*C*_Q_ this work [MHz]	*η* this work	*C*_Q_^[^ ^28^ ^,^ ^36^ ^]^ [MHz]	*η* ^[^ ^28^ ^,^ ^36^ ^]^
KHCO_3_	Site 1	8.6 ± 0.5	6.6	1.495 ± 0.2	0.24	1490	0.25
K_2_CO_3_	Site 1	30.5 ± 0.5	29.2	1050 ± 0.2	0.65	1050	0.65
	Site 2	11.5 ± 0.5	8.8	3260 ± 0.2	0.23	3260	0.25
K_2_CO_3_·1.5H_2_O	Site 1	11.5 ± 0.5	n.a	1000 ± 0.2	0.05	1536	0.86
	Site 2	4.0 ± 0.5	n.a	1450 ± 0.2	0.8	1546	0.63

a) *δ*_iso_, isotropic chemical shift; *C*_Q_, quadrupolar coupling constant; *η*, asymmetry parameter.

Samples **1** (100% KHCO_3_) and **3** (87% KHCO_3_) for the minerals formed by atmospheric CO_2_ captured in aqueous‐ethanol and aqueous KOH solutions consist mostly of KHCO_3_ with resonances (**Table** **4**) with fits that are in good agreement with previous studies of this mineral. In contrast, samples **2** and **4** formed in aqueous‐acetone KOH solutions by atmospheric CO_2_ or by saturation with solid CO_2_ at low temperature, respectively, are close to equal mixtures of KHCO_3_ and K_2_CO_3_·1.5H_2_O, previously characterized by ^39^K NMR by Moudrakovski et al. (simulated ^39^K NMR spectra according to the reported values are shown in Figure S3), and by Bastow.^[^ ^28^ ^,^ ^36^ ^]^

**Table 4:** ^39^K NMR spectral deconvolution for samples **1**–**4** dried at 353 K, and in vacuum for 15 min. No anhydrous K_2_CO_3_ was detected.

Samples	KHCO_3_ [%]	K_2_CO_3_·1.5H_2_O [%]	Disordered K_2_CO_3_ hydrates	KOH [%]
**1**	100	0	0	0
**2**	60	12	27	1
**3**	87	0	13	0
**4**	48	21	31	0

Figure 6 shows a deconvolution for the 39.7 MHz ^39^K spectra for samples **1**–**4** using only the dominant species KHCO_3_ and K_2_CO_3_·1.5H_2_O; the fits do not fully describe the experimental spectrum. Especially for sample **4**, other hydrates are clearly present, but are not stable enough to be identified. This problem arises from, e.g., mixed phases that can be present and equilibrate during temperature phase transitions.^[^ ^37^ ^]^ Furthermore, Gao et al.^[^ ^38^ ^]^ and Liu et al.^[^ ^21^ ^]^ used computational DFT calculations to investigate the adsorption of H_2_O and CO_2_ on the surface of K_2_CO_3_ and the mechanism for promotion of bicarbonate formation for reported forms of K_2_CO_3_. There was a notable dependence of adsorption and reactivity on the exposure of planes and the extent of coverage with H_2_O and CO_2_. For samples **2**, **3**, and **4**, we used a broad ^39^K site with a large distribution of chemical shifts and quadrupolar parameters to account for disordered K_2_CO_3_ phases. It can be estimated that the unidentified disordered K_2_CO_3_ hydrated species represent 31%, 27%, and 13% for samples **4**, **2**, and **3**, respectively (see Figure 6 and Table 4). After the samples were heated at 443 K for 30 min and then kept in vacuum for an additional 15 min, all four samples transformed into anhydrous K_2_CO_3_.

Spectral deconvolution using KHCO_3_ and K_2_CO_3_·1.5H_2_O as main components of the samples **1**–**4** is shown in Table 4. Overall there is a good agreement between the sample speciation determined by NMR and that from Rietveld refinement of the PXRD data shown in Table 1. Both NMR and PXRD agree that sample 1 is 100% KHCO_3_. For samples **2**, **3**, and **4**, NMR gives the percentage of KHCO_3_ as 60%, 87%, and 48%, respectively, whereas for PXRD these percentages are 58%, 100%, and 37%, respectively. Considering the stable and crystalline K_2_CO_3_·1.5H_2_O phase together with disordered K_2_CO_3_ hydrates, the NMR analysis results in similar speciation as that from PXRD, although PXRD is sensitive only to crystalline domains and therefore identifies KHCO_3_ and K_2_CO_3_, whereas NMR, which is capable of detecting local structural environments, shows the presence of hydrated carbonate phases.

Carbon capture technologies can play a role in reducing CO_2_ levels and mitigating temperature rises.^[^ ^39^ ^]^ We have investigated the capture of atmospheric CO_2_ through mineralization in rapid small‐scale reactions of solutions of KOH in water, water/ethanol, water/acetone, and water/acetone/dry ice, followed by evaporation. We focused on identifying the potassium mineral phases by SCXRD, PXRD with Pawley and Rietveld refinements, and high field solid‐state ^1^H (850 MHz, 1 GHz and 1.2 GHz), ^13^C, and ^39^K NMR, including ^1^H, ^1^H‐NOESY experiments to detect H‐bonding environments. Deconvolution of the ^39^K spectra to resolve peaks form individual K^+^ sites was aided by the sharpening of resonances at the highest field (28 T, 56 MHz) due to reduction of second‐order quadrupolar broadening effects for this half‐integer (*I* = 3/2) quadrupolar nucleus.

Although the NMR data do not allow structures to be determined directly, both the ^39^K chemical shifts and the quadrupolar couplings are characteristic of the coordination number and symmetry of the different sites. In this particular case, high field ^39^K MAS NMR allows for differentiation between bicarbonate and carbonate as well as between anhydrous and hydrated carbonate. Further information about H‐bonded protons, the type of bound water and exchange processes is revealed by solid‐state ^1^H and ^13^C MAS NMR.

**Figure 7:**
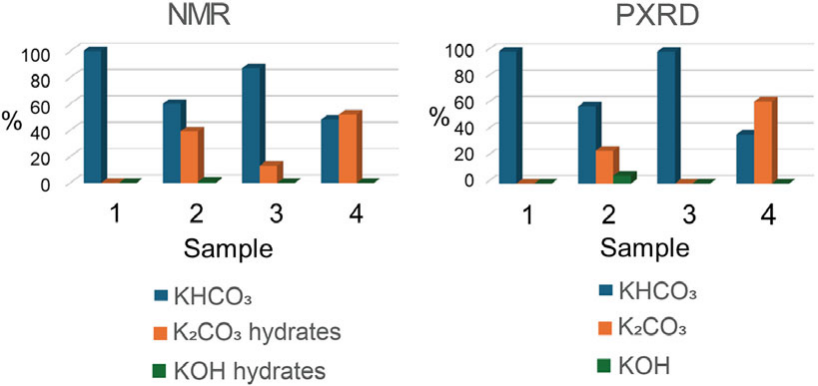
Comparison of the compositions of samples **1**–**4** as determined by NMR and PXRD. The high purity, and hence crystallinity, of sample **1** as KHCO_3_ from capture of atmospheric CO_2_ by aqueous‐ethanolic KOH is apparent. Sample **3** (aqueous KOH) also contains a minor amount of amorphous hydrated K_2_CO_3_.

Whereas NMR can study both the crystalline and amorphous phases of products from CO_2_ capture, SCXRD and PXRD provide information only on the crystalline components. SCXRD identified KHCO_3_ as a product in each of the four reactions studied, with space group *P*2_1_/*n* (transformable to *P*2_1_/*a*). PXRD and NMR confirmed the high purity of KHCO_3_ formed in the aqueous KOH‐ethanol and aqueous KOH reactions. In contrast, the major product from reaction **4** in aqueous KOH‐acetone‐dry ice was K_2_CO_3_, also a significant product in reaction **2** in aqueous KOH‐acetone (**Figure** **7**). A next step would be detailed investigations of the mechanisms of these reactions, for which the subsequent evaporation and drying of products can play important roles. The ability of ^39^K NMR to characterize both the rate and extent of K_2_CO_3_ hydration will be especially useful in future work. As we have shown, no one of the three analytical techniques used is sufficient of provide a detailed characterization of the mineral products, but the combination of them has given greater insights into the composition than any one of them alone.

## 6 Conclusions

Our data demonstrate that a multimodal approach is valuable for providing a detailed picture of the various phases present in these mineral products, although in some cases the challenge of identifying other minor phases remains. These findings contribute to attempts to discover practical, low‐energy routes to add value to captured CO_2_ suitable for industrial mineral feedstocks and pave the way for optimizing solvent systems and crystallization conditions for large‐scale carbon mineralization.

## Supporting Information

The SI contains details of the syntheses, TGA of K_2_CO_3_ and experimental and computational studies by SCXRD, PXRD, and solid‐state NMR. Tables S1 and S2 contain XRD parameters, Table S3 Pawley refinement for PXRD, Table S4 CASTEP calculations of ^1^H, ^13^C and ^39^K NMR parameters, Figures S1 ^13^C, S2, ^1^H NOESY, S3, S4, S5 and S7 39.7 and 56 MHz ^39^K NMR spectra, and Figure S6 X‐ray structures of KHCO_3_, as well as additional references. Additional single crystal XRD data have been deposited in CCDC 2456432‐2456435, and PXRD data in https://wrap.warwick.ac.uk/192193/.

## Conflict of Interest

The authors declare no conflict of interest.

## Author Contributions

**Ehsan Ezzatpour Ghadim** conceptualized the experiments, synthesized samples **1**–**4**, analyzed them by PXRD, and refined and interpreted the data. **Rodrigo S. Corrêa** collected, analyzed, and interpreted the SCXRD data. **Dinu Iuga** and **Stephanie Bachmann** designed the NMR experiments, and recorded and interpreted the data. **Peter Sadler** and **Joanna F. Collingwood** assisted with conceptualization of the experiments. **Peter J. Sadler** coordinated the analytical measurements and compiling of the paper. All authors were involved with the drafting of the paper as well as reviewing and editing the final submission.

## Supporting information

Supplementary Material

## Data Availability

The data that support the findings of this study are available via the supplementary material provided for this article.
