# Supplementary material for: Mineral Speciation for CO2 Captured by Potassium Hydroxide
Source: ChemistryOpen. 2025 Oct 14;15(4):e202500376. doi: 10.1002/open.202500376 (PMC13052176; doi:10.1002/open.202500376)
Supplement: Supplementary file 1 — Supplementary Material [file OPEN-15-e202500376-s001.pdf]

Supporting Information

Mineral Speciation for CO_2 captured by Potassium Hydroxide

Ehsan Ezzatpour Ghadim, Stephanie Bachmann, Rodrigo S. Correa, Dinu Iuga, Joanna F. Collingwood, and Peter J. Sadler

[a] *Ehsan Ezzatpour Ghadim, Joanna F. Collingwood*

School of Engineering, University of Warwick, Coventry CV4 7AL, UK

E-mail: ehsan.ezzatpour-ghadim.3@warwick.ac.uk; Email: J.F.Collingwood@warwick.ac.uk

b] *S. Bachmann, D. Iuga*

Department of Physics, University of Warwick, Coventry, CV4 7AL, UK

E-mail: Stephanie.Bachmann@warwick.ac.uk; E-mail: D.Iuga@warwick.ac.uk

[c] *R. S. de Souza Corrêa, P. J. Sadler*

Department of Chemistry, University of Warwick

Gibbet Hill Road, Coventry CV4 7AL, UK

E-mail: Rodrigo.De-Souza-Correa@warwick.ac.uk; E-mail: P.J.Sadler@warwick.ac.uk

[d] *Department of Chemistry, Federal University of Ouro Preto, Ouro Preto, 35402-136, Minas Gerais, Brazil*

Table of Contents

S1. Materials S3

S1.1 Chemicals S3

S1.2 Syntheses S4

S2.1 Single crystal X-ray diffraction (SCXRD) S5

Table S1. Crystal, data collection and refinement for potassium bicarbonate, $KHCO_3$ present in samples **1 – 4**. S5

Table S2. Selected bond lengths [Å] and angles [°] for $KHCO_3$ in samples **1** - **4**. S6

S2.2. Powder X-ray diffraction (PXRD) S8

Table S3: Unit cell parameters determined by Pawley refinement compared to powder XRD patterns for samples **1**-**4** with various phases. S9

S2.3 Solid-state NMR spectroscopy S9

Table S4. Summary of CASTEP/GIPAW[8-10] calculations of ^{1}H, ^{13}C and ^{39}K NMR parameters based on literature known crystal structures. S11

Figure S1. ^{13}C CPMAS and DPMAS NMR spectra of samples **1 - 4** S13

Figure S2. 1 GHz ^{1}H-^{1}H 2D NOESY NMR spectrum of sample **2** with a mixing time of 800 ms and assignment of peaks S14

Figure S3. Simulated ^{39}K NMR spectrum of K_2CO_3 and $KHCO_3$ fitted according to the values reported by Moudrakovski et al. S15

Figure S4. Experimental and simulated ^{39}K NMR spectra of anhydrous K_2CO_3. S15

Figure S5. Experimental and simulated 56 MHz ^{39}K NMR spectra of partly rehydrated K_2CO_3. S16

Figure S6 Potassium sites in X-ray crystal structures of (A) anhydrous K_2CO_3, and (B) K_2CO_3 1.5H_2O S17

Figure S7. Comparison of observed and simulated 56 MHz and 39.7 MHz ^{39}K NMR spectra of partially rehydrated K_2CO_3 S18

References S19

S1. Materials

S1.1 Chemicals

Absolute ethanol (99%, CAS 64-17-5) and KOH (CAS 1310-58-3) were purchased from Fisher Scientific, anhydrous acetone (CAS 67-64-1, 99.98% purity) from VWR, K_2CO_3 from BDH Limited, and dry ice from BOC. Deionized water (18 MΩ·cm) was obtained from a Simplicity UV ultrapure water system (Millipore) and used in all aqueous procedures. K_2CO_3 is hygroscopic.

A sample of the K_2CO_3 used was dried for 15 min in an oven at 373 K, then placed in a vacuum for 15 min (similar to the procedure for the NMR sample), and then the water content was determined by thermogravimetric analysis (TGA) using a Mettler Toledo TGA/DSC instrument, over the temperature range of 303 – 573 K (30–300 °C), with a N_2 gas flow of 50 mL/min.

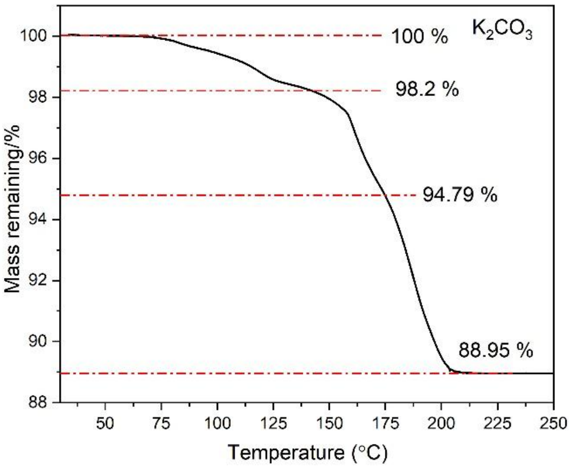

The data suggest that the sample contains both a small amount of adsorbed water (loss over 80-120 ^oC) and water of crystallisation (hydrate form; loss over 150-210 ^oC), and that the major phase present (ca. 62%) is $K_2CO_31.5H_2O$.

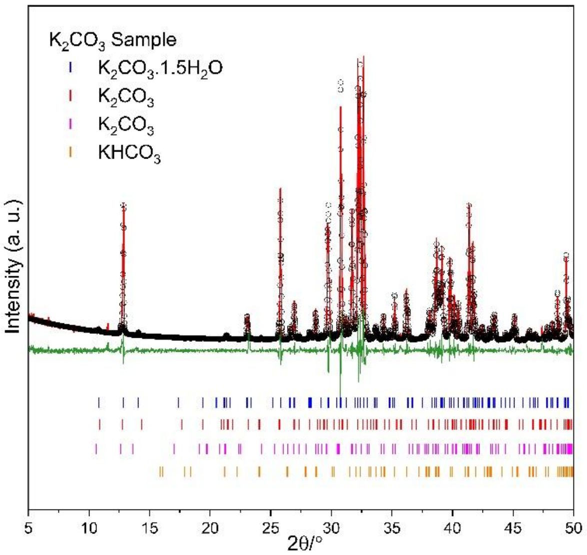

The PXRD on a similar sample fitted by GSAS (above) showed a a similar proportion of the hydrated form: $K_2CO_3.1.5H_2O$ (65.5%), K_2CO_3 (16.7%, with ICSD 10191 and 66943), and $KHCO_3$ (17.8%).

S1.2 Syntheses

A stock solution was prepared by dissolving 280.5 g of solid KOH in 500 mL of deionized water in 500 mL Nalgene® bottle, and sonicated to aid dissolution to give a 10 M solution, which was the capped. This was used in each of the syntheses below. All syntheses were carried out within the airflow of a laboratory fume hood.

Sample 1: KOH/water/ethanol

Sample **1** was prepared by mixing 15 mL KOH (10 M, prepared above) with 15 mL of deionized water and 15 mL of ethanol in a 50 mL glass beaker and then heating to 353 K on a hotplate. Then, the solution was allowed to cool at ambient temperature (292 K), enabling CO_2 from the air to react with the mixture. The initial pH of ca. 14 decreased to ca. 10 (measured using VMR Rota wide range pH 1-14 paper). A small aliquot of this solution was transferred to a glass slide, where crystals formed in the air after 30 min at ambient temperature (292 K). SCXRD was studied using these crystals. The remaining solution was allowed to evaporate to dryness over 2 days. PXRD and NMR were used to study this dried sample.

Sample 2: KOH/water/acetone

Sample **2** was prepared by mixing 15 mL of KOH (10 M) with 15 mL of deionized water and 15 mL of anhydrous acetone and heated on a hotplate at 333 K in a 50 mL beaker without mixing. After reaching 333 K, the solution was allowed to evaporate at ambient temperature in the air (292 K) until the crystals appeared. The initial pH of 14 decreased to 10. A small amount of the mother liquor was poured onto a glass slide, and crystals formed within 20 min at ambient temperature (292 K) in the air. The remaining solution in the beaker was allowed to evaporate overnight (ca. 12 h). SCXRD was used to analyze the crystals, whilst the dried samples were studied by PXRD and solid-state NMR.

Sample 3 : KOH/water

Sample **3** was prepared by heating 15 mL of KOH (10 M) and 15 mL of deionized water to 353 K in a 100 mL beaker on a hotplate. Once the solution reached 353 K, it was left to cool down to ambient temperature (292 K), allowing CO_2 in the air to react with the solution. A small portion of the solution was transferred to a glass slide and dried for 90 min. The initial pH of 14 decreased to 10 as measured by pH paper. Crystal formation was observed after 95 min, and these crystals were used for study by SCXRD. The remaining solution in the beaker dried within 2 days. SCXRD was used to analyze the crystals on the slide, while PXRD and solid-state NMR were used to study the dry samples.

Sample 4: KOH/water/acetone/dry ice

Sample **4** was prepared by adding 15 mL of KOH (10 M), 15 mL of deionized water, and 15 mL of acetone to a 100 mL of beaker at 333 K on a hotplate. After the solution began to boil at 333 K, heating was stopped; the beaker was removed from from the hotplate, and immediately 40 g of dry ice was added, lowering the temperature to ca. 271 K. After adding the dry ice, crystals rapidly appeared at the bottom of the beaker after 15 min at 271 K, and the pH decreased from ca. 14 initially to ca. 7, as measured by pH paper. 2 mL of the solution containing crystals was transferred to a 3 mL Eppendorf tube for SCXRD, and the remaining sample in the beaker was recovered by centrifugstion in 5 min (5000 rpm) and dried at ambient temperature (292 K) in the air under the fume hood.

Sample preparation for analysis

For SCXRD, 2 mL of the mother liquor containing the crystals of each sample was added to a 3 mL plastic Eppendorf tube.

For PXRD, the same procedure as above was used, and 25 mg of each sample was used for the measurements at ambient temperature.

For solid state NMR**,** 100 mg of each sample was used after drying in an oven at 353 K for 15 min and storing under vacuum for 15 min. For NMR and PXRD analysis the dried samples were ground with a pestle and mortar. Dehydrated K_2CO_3 was prepared for some NMR samples by additional drying at 353 K and 448 K (as noted in the text).

S2.1 Single crystal X-ray diffraction (SCXRD)

Single crystals from samples **1-4** were analyzed by X-ray diffraction. Suitable crystals were selected and data collection was carried out on an Oxford Diffraction Synergy-S at low temperature (100 K), using Cu-radiation source [CuKα = 1.54060 Å]. The instrument is equipped with a large theta coverage detector HyPix-Arc 100° and a four-circle system. For data collection and processing, the *CrysAlisPro* was used.[1]

The crystal structures were solved by direct methods using SHELXS. Initially the structures were solved in the $P2_1/n$ space group, subsequently the transformation matrix below was used to transform the space group from $P2_1/n$ to $P2_1/a$:

$$\begin{matrix} -1 & 0 & 1 \\ 0 & 1 & 0 \\ 1 & 0 & 0 \end{matrix}$$

Thus, the $P2_1/a$ space group was considered, and the structures were refined by full-matrix least squares against F^2, using SHELXL-97.[2] Anisotropic displacement parameters were used for all non-H atoms, meanwhile the H-atom was located in a Fourier map and refined considering $U_{iso}(H) = 1.5U_{eq}(O)$. Mercury 2024.2.0 software[3] was used for analysis of intra and intermolecular contacts, as well as to generate all crystal structure representations (Figure 1). Details of crystal structure refinement are in Table S1 and selected bond lengths and bond angles in Table S2.

Table S1. Crystal, data collection and refinement for potassium bicarbonate, $KHCO_3$ present in samples **1–4**.

	Sample **1**	Sample **2**	Sample **3**	Sample **4**
Empirical formula	$KHCO_3$			
Formula weight	100.12			
Temperature (K)	100(2)			
Crystal system	Monoclinic			
Space group	$P2_1/a$			
Unit cell dimensions (Å)	*a* = 15.1162(6)	15.1224(5)	15.1231(5)	15.1229(5)
	b = 5.6021(3)	5.6059(2)	5.6037(2)	5.6058(2)
	c = 3.6587(2)	3.6599(1)	3.6589(1)	3.6596(1)

	β= 104.039(4)	104.059(4)	104.063(3)	104.051(3)
Volume (Å^3)	300.57(3)	300.973(18)	300.781(17)	300.963(17)
Z	4	4	4	4
Density (Mg/m^3)	2.212	2.209	2.211	2.210
μ (mm^{-1})	13.844	13.825	13.834	13.826
F(000)	200	200	200	200
Crystal size (mm^3)	0.157 x 0.055 x 0.043	0.188 x 0.097 x 0.065	0.282 x 0.112 x 0.080	0.410 x 0.150 x 0.140
θ range for data collection	6.035 to 78.848°	8.466 to 75.924°	6.033 to 73.639°	6.033 to 73.620°
Index ranges	-18≤h≤17, -6≤k≤2, -4≤l≤4	-11≤h≤18, -6≤k≤6, -4≤l≤4	-18≤h≤13, -6≤k≤6, -4≤l≤4	-18≤h≤17, -6≤k≤6, -4≤l≤4
Reflections collected	1504	2502	2481	2494
Independent reflections	585 [R(int) = 0.0207]	594 [R(int) = 0.0199]	593 [R(int) = 0.0300]	585 [R(int) = 0.0221]
Completeness to theta (%)	99.8	99.8	100.0	99.8
Data / restraints / parameters	585 / 0 / 46	594 / 0 / 46	593 / 0 / 46	585 / 0 / 46
Goodness-of-fit on F^2	1.081	1.086	1.117	1.052
Final *R* indices [I>σ(I)]	*R1* = 0.0234, *wR2* = 0.0640	0.0184, 0.0494	0.0239, 0.0674	0.0228, 0.0637
R indices (all data)	*R1* = 0.0250, *wR2* = 0.0653	0.0187, 0.0495	0.0243, 0.0678	0.0230, 0.0639
$\rho_{max.}/\rho_{min}$ (e.Å^{-3})	0.258 / -0.303	0.244 / -0.246	0.417 / -0.427	0.292 / -0.396
CSD Number	2456432	2456433	2456434	2456435

Table S2. Selected bond lengths [Å] and angles [°] for $KHCO_3$ in samples **1**-**4**.

Bond	*Sample 1*	*Sample 2*	*Sample 3*	*Sample 4*
C1-O1	1.357(2)	1.3563(17)	1.358(2)	1.3597(18)
C1-O2	1.250(2)	1.2507(18)	1.246(2)	1.246(2)
C1-O3	1.261(2)	1.2646(18)	1.264(2)	1.264(2)
K1-O1 ^{i}	2.8694(13)	2.8708(11)	2.8700(12)	2.8722(11)
K1-O2 ^{ii}	2.6857(13)	2.6848(10)	2.6873(13)	2.6868(12)
K1-O3	2.7176(13)	2.7196(10)	2.7190(12)	2.7175(11)
K1-O1 ^{iii}	2.7777(13)	2.7792(10)	2.7792(12)	2.7783(11)

K1-O3 iv	2.7807(13)	2.7830(11)	2.7818(12)	2.7831(11)
K1-O2 v	2.8327(14)	2.8319(11)	2.8315(13)	2.8324(12)
K1-O2 iii	2.8881(14)	2.8915(11)	2.8910(12)	2.8919(11)
K1-O2	3.0105(14)	3.0113(11)	3.0100(12)	3.0108(11)
K1...K1 vi	3.6587(2)	3.65989(10)	3.65887(10)	3.65959(10)
Bond Angle				
O1-C1-O2	115.79(17)	115.98(12)	116.06(14)	115.95(13)
O1-C1-O3	118.32(17)	118.27(12)	118.05(15)	118.08(15)
O2-C1-O3	125.89(18)	125.75(13)	125.89(16)	125.97(15)

Symmetry codes: i) -x+3/2,y-1/2,-z+3 ; ii) -x+3/2,y+1/2,-z+2 ; iii) -x+3/2,y-1/2,-z+2 ; iv) -x+3/2,y+1/2,-z+3, v) x,y,z-1 ; vi) x,y,z+1

S2.2. Powder X-ray diffraction (PXRD)

PXRD measurements were carried out on an Analytical Empyrean diffractometer using copper Kα1/2 radiation with an average wavelength of 1.5418 Å, at ambient temperature. The diffraction data were recorded over a 5–50 °2θ range.

The PXRD pattern profiles were fitted with the General Structure Analysis System II software (GSAS-II version 5806, python 3.13.3 64-bit) to extract the unit cell parameters.[4] Pawley and Rietveld refinements were used to analyze the PXRD patterns for the presence of different phases of $KHCO_3$ (ICSD = 2327), KOH (ICSD = 47114), and K_2CO_3 (ICSD = 662).

Table S3. Unit cell parameters determined by Pawley refinement for powder XRD patterns of samples **1-4** with various phases.

Sample	Phase	Space group	*a* (Å)	*b* (Å)	*c* (Å)	*β*	V (Å³)
**1**	$KHCO_3$	$P2_1/a$	3.71059	5.62496	14.67294	90.487	306.241
	$KHCO_3$	$P2_1/a$	3.73973	5.61572	14.70691	90.006	308.864
**2**	K_2CO_3	*C2/c*	5.70701	9.78386	6.98214	96.638	387.246
	KOH	$I4_1/amd$	6.73637	---	12.88082	---	584.515
**3**	$KHCO_3$	$P2_1/a$	3.68448	5.60977	14.62575	89.543	302.291
**4**	$KHCO_3$	$P2_1/a$	3.7283	5.58706	14.74627	89.735	307.165
	K_2CO_3	*C2/c*	5.5434	10.08522	7.08324	94.923	394.537

S2.3 Solid-state NMR spectroscopy

NMR samples, data acquisition and processing

The samples for NMR analysis were dried for 15 min 15 min in an oven at 373 K, then placed in a vacuum for 15 min, and ground before packing into a 4 mm ZrO_2 rotor. To obtain anhydrous K_2CO_3 , $K_2CO_3 \cdot 1.5H_2O$ was dried for 15 min at 448 K and stored in vacuum for additional 15 min.

The spectra were recorded on an 850 MHz (for ^1H) spectrometer (20.0 T) using a 4 mm HX probe and a spinning frequency of 10 kHz. All measurements were acquired at room temperature. The magic angle was set with KBr. L-alanine was used to optimized the ^1H 90° pulse of 3.4 µs. For all ^1H spectra an interscan delay of 5 s was used. For the ^{13}C CP conditions a ramp of 70 to 100 and a contact time of 1 ms was used. 214 MHz ^{13}C DPMAS spectra were acquired using a ^{13}C 90° pulse length of 4 µs, an interscan delay of 10 s and high power decoupling of the protons during acquisition.

For sample **2**, a ^1H-^1H NOESY with a mixing time of 800 ms, 6 s recycle delay, 16 scans and 502 increments, and a ^1H spectrum with 8 scans and 20 s recycle delay was recorded on a 1 GHz spectrometer (23.5 T) using the 1.3 HX probe and a spinning frequency of 60 kHz. This ^1H spectrum was referenced on beta-AspAla. The remaining ^1H and ^{13}C spectra were referenced using alanine. ^{13}C chemical shifts were referenced to the carbonyl carbon of L-alanine at $\delta(^{13}C)$ = 177.8 ppm, with respect to tetramethylsilane (TMS), corresponding to the methylene carbon of adamantane at $\delta(^{13}C)$ = 38.48 ppm.[5] whereas ^1H spectra were referenced on the signal of CH of alanine at δ(1H) = 3.5 ppm.

39.7 MHz ^{39}K measurements were recorded by using the double frequency switch (dfs)[6] from 145 to 1000 kHz with echo, a 90° pulse of 7 µs, an interscan delay of 2 s and a spinning rate of 12 kHz. The 56,1 MHz ^{39}K spectra of K_2CO_3 and $K_2CO_3 \cdot 1.5H_2O$ shown in Figure 4 were measured on the 28.2 T (1.2 GHz ^1H) spectrometer. ^{39}K spectra were referenced by using the ^{39}K shift of KBr at 55.4 ppm.[7] Further evaluation of the ^{39}K spectra was done with the sola module of the Bruker Software version TopSpin 4.4.1.

Chemical shift assignments were supported by CASTEP/GIPAW[8-10] calculations of literature structures. The academic version of CASTEP 22.1 was used.

CASTEP/GIPAW calculations

Chemical shift assignment was supported by CASTEP/GIPAW[8-10] calculations of literature known structures. Crystal structures were downloaded from the Cambridge Structural Database.[11] To calculate the exchange-correlation potential PBE[12] was used. The cut-off energy was set to 800 eV. The following table gives an overview about all calculated structures with the CCDC identifier and the calculated values, respectively. Furthermore, it needs to be considered, that for some structures constraints were used. The chemical shifts of ^{1}H, ^{13}C and ^{39}K were calculated by using the calculated shielding tensors and a reference value of δ_{ref} = 29.6[13], 168.1[13] and 1210 (estimated), respectively.

The calculations were made at a temperature of 0 K and do not consider movement in the system. This can lead to deviations from the experimental data especially as the systems here are salts.

Table S4. Summary of CASTEP/GIPAW[8-10] calculations of ^{1}H, ^{13}C and ^{39}K NMR parameters based on known literature crystal structures. Where commented, the calculation of the NMR parameters was either done after geometry optimization where the position of all atoms was not fixed (geom), or with fixing all atoms (fix all) except the ^{1}H atoms.

**Crystal structure**	**CCDC database identifier**	**Com-ment**	**Shielding tensor ^{13}C**	**+Reference. chem shift / ppm**	**Shielding tensor ^{1}H**	**+Reference. chem shift / ppm**	**Shielding tensor ^{39}K**	**+ Reference. chem shift / ppm**	**C_Q / MHz**	**η_Q**
$K_2CO_3\cdot$ 1.5 H_2O	-	Rhodes et al[14]	1.34	166.8	24.2	5.4	1201.76	8.24	-1.427	0.62
			-	-	23.1	6.5	1200.33	9.67	-1.707	0.51
			-	-	21.77	7.83	1216.84	-6.84	-1.354	1
			-	-	-	-	1201.77	8.23	-1.354	1
$KHCO_3$		Rhodes et al[14]	8	160.1	15.78	13.82	1211.07	-1.07	1.229	0.29
K_2CO_3, ($P2_1/c$)	CCDC 1628983[15]	geom	-0.05	168.2	-	-	1209.41	0.59	1.49	0.6
			-	-	-	-	1180.66	29.34	3.5	0.25
$KHCO_3$ ($P2_1/a$)	CCDC 1592020[16]	geom	8.04	160.1	15.46	14.14	1211.19	-1.19	1.481	0.53
$K_2CO_3\cdot$ 1.5 H_2O ($C2/c$)	CCDC 2273323 [17]	geom	1.51	166.6	24.5	5.1	1197.99	12.01	-1.378	0.02
			-	-	23.21	6.39	1200.7	9.3	-1.75	0.43
			-	-	22.14	7.46	1212.95	-2.95	1.404	0.87
$K_2C_2O_6$ ($P2_1/c$)	CCDC 1727671 [18]	geom	12.77	155.3	-	-	1211.01	-1.01	-1.692	0.88
			13.16	154.9	-	-	1221.85	-11.85	0.63	0.68
$KOH\cdot$ 2 H_2O ($I4_1/amd$)	CCDC 1608305 [19]	fix all, except ^{1}H	-	-	14.16	15.4437	1201.24	8.7598	-0.7129	0.7671
			-	-	22.25	7.3521	-	-	-	-
$KHCO_3$ ($C2/m$)	CCDC 1676988 [20]	fix all, except ^{1}H	15.6135	152.49	14.19	15.4143	1211.57	-1.5734	1.4991	0.7145
			7.1493	160.95	14.76	14.8354	1209.73	0.268	1.91	0.7104
		geom	8.0278<br>8.0397	160.0722<br>160.0603	15.4035<br>15.4238	14.1965<br>14.1762	1210.497<br>1210.341	-0.497<br>-0.341	1.3443<br>1.4205	0.6297<br>0.6847

$CH_7K_2O_{11}$ (*C*2/*c*)	CCDC 1733732 [21]	fix all, except ^{1}H	10.6737	157.4	-	-	1234.64	-24.6417	-1.069	0.9632
			-	-	-	-	1214.736	-4.7357	-1.1719	0.2281
		geom	7.0088	161.09	16.3467<br>18.1574<br>17.7281<br>14.8533<br>15.3398<br>17.8128<br>19.5374	13.2533<br>11.4426<br>11.8719<br>14.7467<br>14.2602<br>11.7872<br>10.0626	1235.334<br>1215.858	-25.334<br>-5.858	1.0817<br>-1.1238	0.6469<br>0.2191
K_2CO_3 (*P*6_3/*mmc*)	CCDC 1728885 [22]	Geom (CO_3 units rotating)	-2.094	170.2	-	-	1211.483	-1.4831	6.80	-
			-	-			1186.396	23.6042	-2.20	-
		no geom.	-	163.9			1167.20	42.7977	8.2256	-
			-	-			1196.532	13.468	-1.8954	-

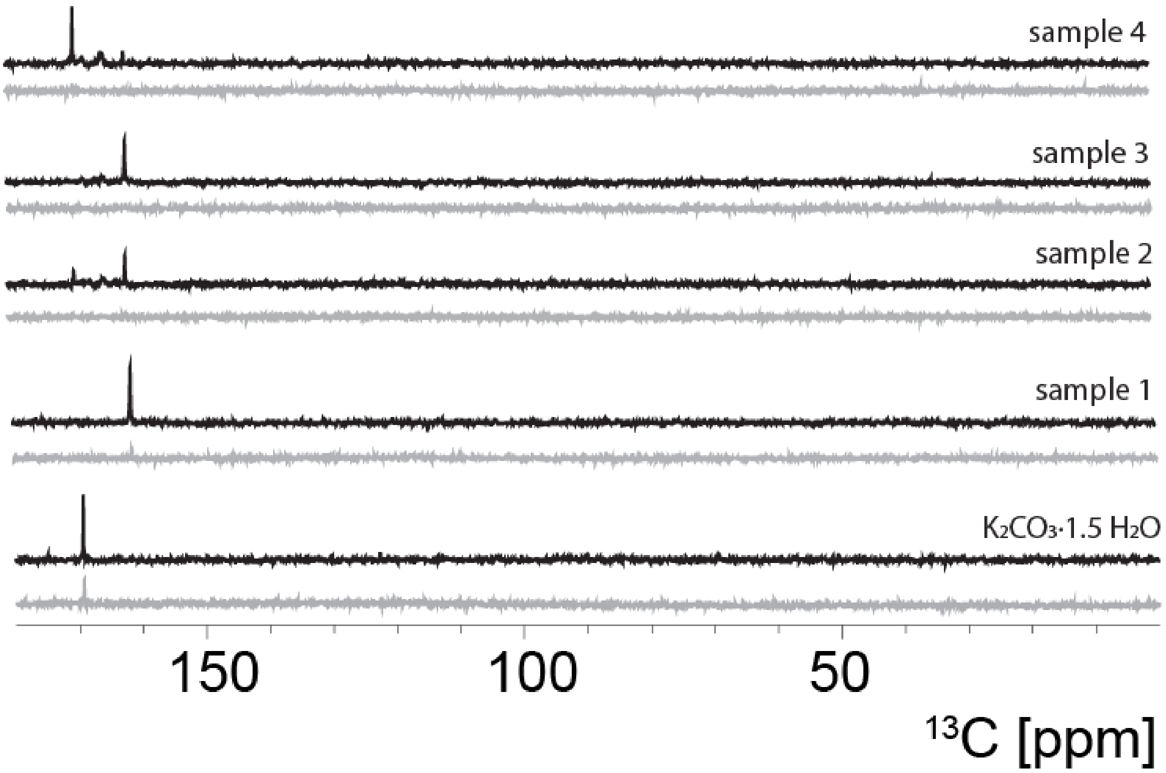

Figure S1. 213 MHz ^{13}C CPMAS (black) and DPMAS (grey) NMR spectra of samples **1** – **4** and $K_2CO_3 \cdot 1.5\ H_2O$. The ^{13}C CPMAS spectra were recorded with 64 scans and an interscan delay of 10 s. The ^{13}C direct excitation (DE) spectra were recorded with 24 scans and an interscan delay of 10 s.

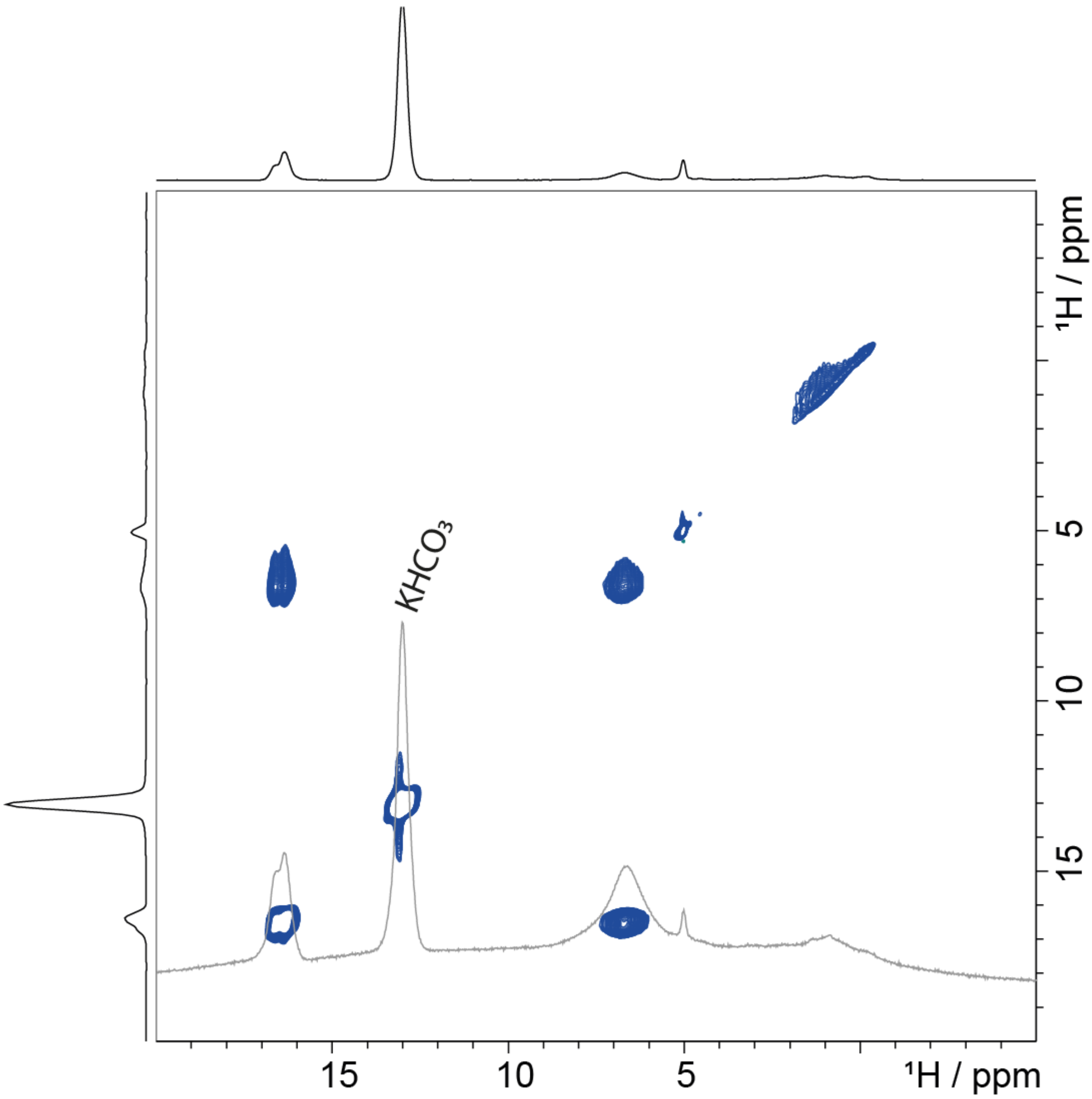

Figure S2. 1 GHz ^{1}H-^{1}H 2D NOESY NMR spectrum of sample **2** with a mixing time of 800 ms (for further assignment of peaks see Table 2).

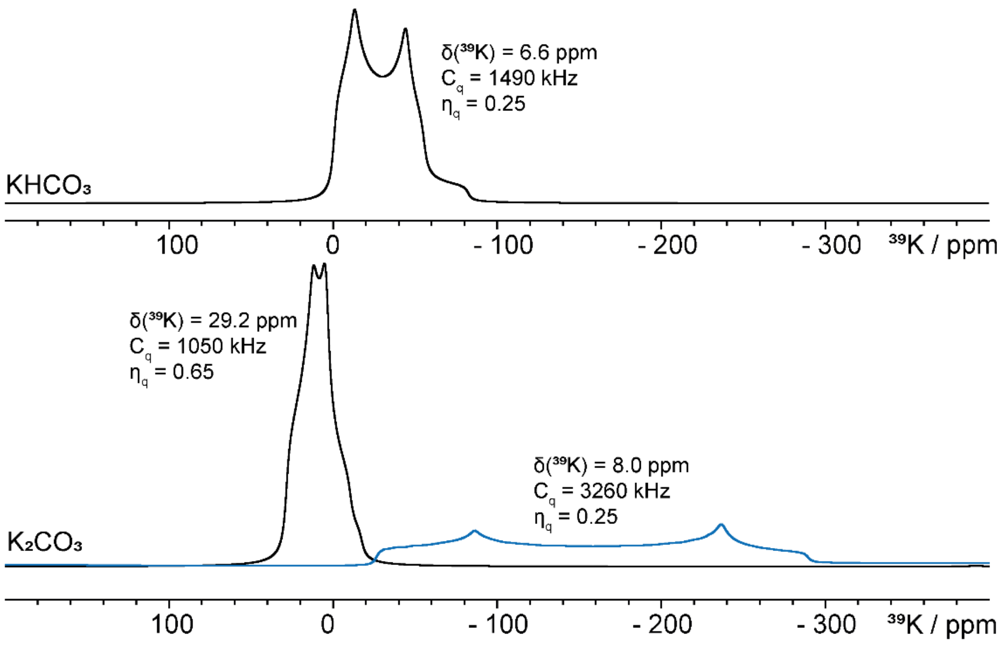

Figure S3. Simulated 39.7 MHz ^{39}K NMR spectra of K_2CO_3 and $KHCO_3$ fitted according to the values reported by Moudrakovski et al.[7] For K_2CO_3, there are two distinct ^{39}K sites shown in blue and black.

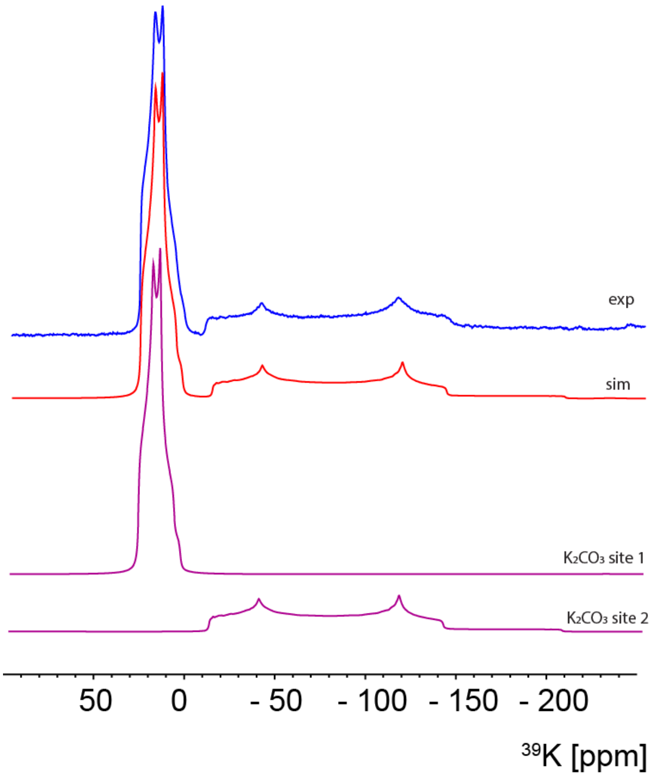

Figure S4. Experimental and simulated 56 MHz ^{39}K NMR spectra of anhydrous K_2CO_3..

The experimental spectrum is shown in blue (see Fig 4C). The simulated spectrum in red is the sum of the two sites (purple) of anhydrous K_2CO_3 with the values given in Table 3.

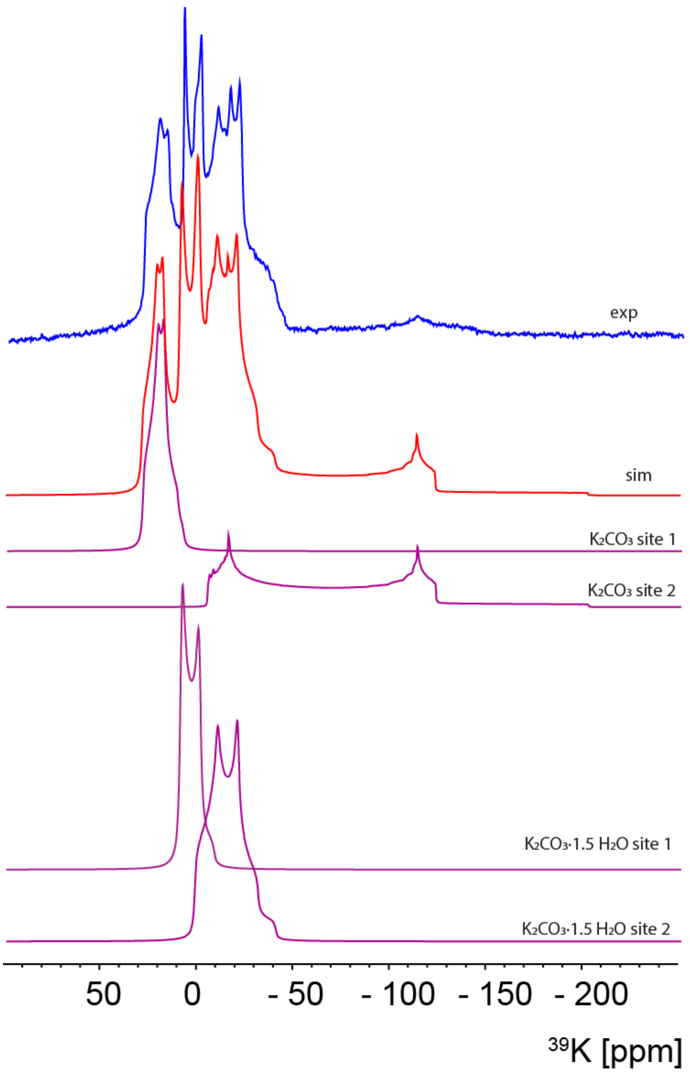

Figure S5. Experimental and simulated 56 MHz ^{39}K NMR spectra of partly rehydrated K_2CO_3. The experimental spectrum is shown in blue (see Fig 4D). The simulated spectrum in red is the sum of the two sites of anhydrous K_2CO_3 (top, purple lines) with the values give in Table 3 and two sites of the $K_2CO_3 \cdot 1.5H_2O$ (bottom purple lines) with the values given in Table 3.

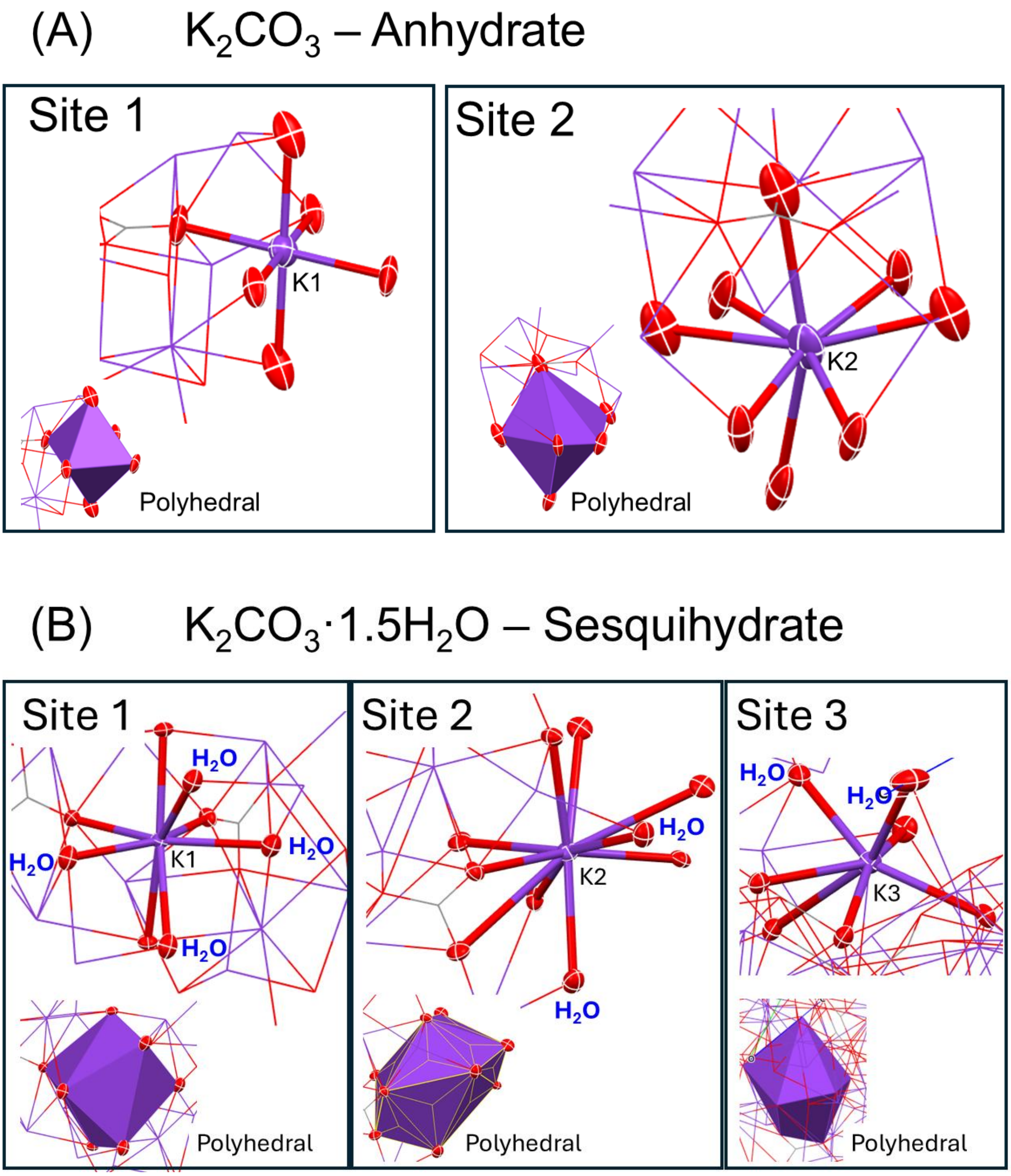

Figure S6. Potassium sites in X-ray crystal structures of (A) anhydrous K_2CO_3, [23] and (B) K_2CO_3 $1.5H_2O$.[24]

In both the anhydrate (Figure S6A) and in the sesquihydrate (1.5 H_2O) (Figure S6B) forms of K_2CO_3 the K^+ atoms are located in distinct sites. In the anhydrate, one K^+ has an octahedral geometry coordinated to O-atoms of carbonate, while the other K^+ is surrounded by eight-oxygen atoms of carbonates. The crystal structure of K_2CO_3 $1.5H_2O$ exhibits three distinct

sites for K^+, with different coordination modes involving oxygens of water and carbonate. Two K^+ atoms are located on a two-fold axis within the channels and another located within the H-bonded chains that link them.

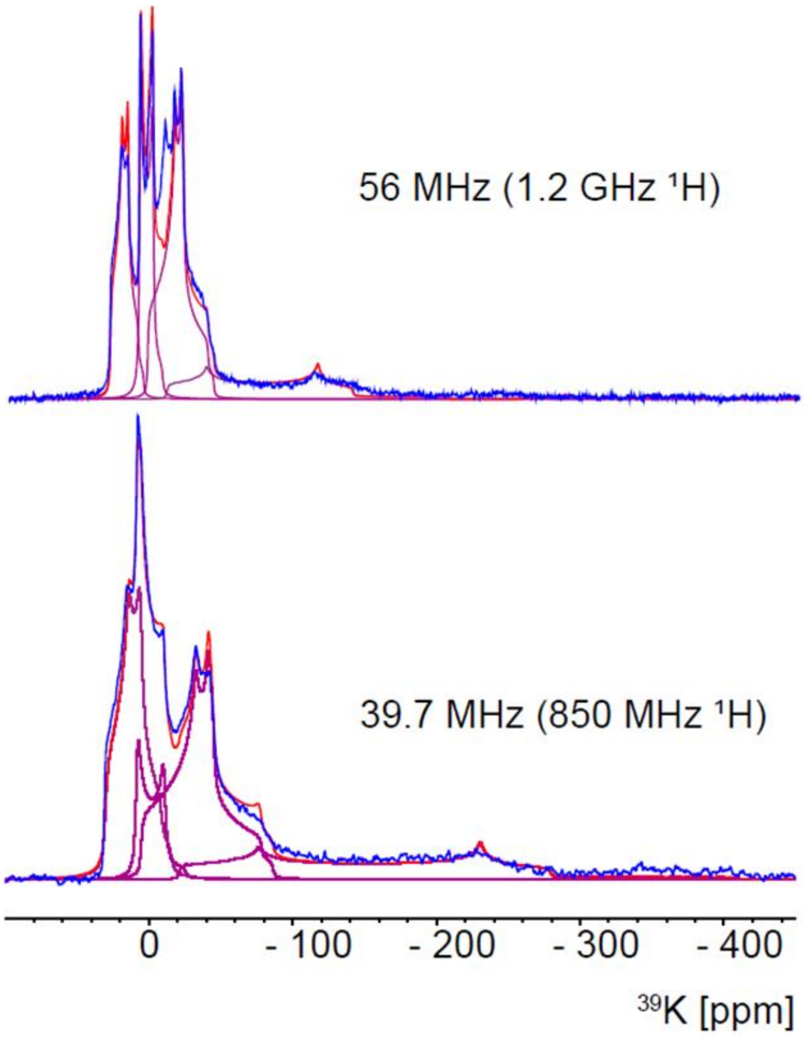

Figure S7. Comparison of observed (blue) and simulated (red) 56 MHz (top) and 39.7 MHz (bottom) ^{39}K NMR spectra of partially rehydrated K_2CO_3 (see also Figures 4D and S5). The ability of the higher field (28 T vs 20 T, 850 MHz vs 1.2 GHz for 1H) to resolve the two K^+ sites in each of anhydrous K_2CO_3 and $K_2CO_3 \cdot 1.5H_2O$ is evident. The simulated spectra in red are the sum of the two sites in anhydrous K_2CO_3 and the two sites in $K_2CO_3 \cdot 1.5H_2O$ using values of parameters given in Table 3 (purple lines in simulated spectra in Fig 6).

References

[1] U. Oxford Diffraction. CrysAlisPro Software System; Rigaku Corporation: Oxford, 2015.

[2] G. M. Sheldrick, "SHELXT: integrating space group determination and structure solution" *Acta Crystallogr., Sect. A:Found. Crystallogr.* **2015**, *71*, 3-8.

[3] C. F. Macrae, I. Sovago, S. J. Cottrell, P. T. A. Galek, P. McCabe, E. Pidcock, M. Platings, G. P. Shields, J. S. Stevens, M. Towler, P. A. Wood, "Mercury 4.0: from visualization to analysis, design and prediction" *J. Appl. Crystallogr.* **2020**, *53*, 226-235.

[4] B. H. Toby, R. B. Von Dreele, "GSAS-II: the genesis of a modern open-source all purpose crystallography software package" *J. Appl. Crystallogr.* **2013**, *46*, 544-549.

[5] C. R. Morcombe, K. W. Zilm, "Chemical shift referencing in MAS solid state NMR" *J. Magn. Reson.* **2003**, *162*, 479-486.

[6] D. Iuga, A. P. M. Kentgens, "Influencing the satellite transitions of half-integer quadrupolar nuclei for the enhancement of magic angle spinning spectra" *J. Magn. Reson.* **2002**, *158*, 65-72.

[7] I. L. Moudrakovski, J. A. Ripmeester, "^{39}K NMR of solid potassium salts at 21 T: effect of quadrupolar and chemical shift tensors" *J. Phys. Chem. B* **2007**, *111*, 491-495.

[8] S. J. Clark, M. D. Segall, C. J. Pickard, P. J. Hasnip, M. I. J. Probert, K. Refson, M. C. Payne, "First principles methods using CASTEP" *Z. Kristallogr.* **2005**, *220*, 567-570.

[9] C. J. Pickard, F. Mauri, "All-electron magnetic response with pseudopotentials: NMR chemical shifts" *Phys. Rev. B* **2001**, *63*, 245101.

[10] J. R. Yates, C. J. Pickard, F. Mauri, "Calculation of NMR chemical shifts for extended systems using ultrasoft pseudopotentials" *Phys. Rev. B* **2007**, *76*, 024401.

[11] C. R. Groom, I. J. Bruno, M. P. Lightfoot, S. C. Ward, "The Cambridge Structural Database" *Acta Crystallogr., Sect. B:Struct. Sci., Cryst. Eng. Mater.* **2016**, *72*, 171-179.

[12] J. P. Perdew, K. Burke, M. Ernzerhof, "Generalized Gradient Approximation Made Simple" *Phys. Rev. Lett.* **1996**, *77*, 3865-3868.

[13] G. N. Reddy, D. S. Cook, D. Iuga, R. I. Walton, A. Marsh, S. P. Brown, "An NMR crystallography study of the hemihydrate of 2', 3'-O-isopropylidineguanosine" *Solid State Nucl. Magn. Reson.* **2015**, *65*, 41-48.

[14] B. J. Rhodes, L. L. Schaaf, M. E. Zick, S. M. Pugh, J. S. Hilliard, S. Sharma, C. R. Wade, P. J. Milner, G. Csanyi, A. C. Forse, "^{17}O NMR Spectroscopy Reveals CO(2) Speciation and Dynamics in Hydroxide-Based Carbon Capture Materials" *ChemPhysChem* **2025**, *26*, e202400941.

[15] Y. Idemoto, J. W. Richardson, N. Koura, S. Kohara, C.-K. Loong, "CCDC 1628983: Crystal structure of (LixK1 − x)2CO3 (x = 0, 0.43, 0.5, 0.62, 1) by neutron powder diffraction analysis; Cambridge Crystallographic Data Centre." *J. Phys. Chem. Solids* **1998**, *59*, 363-376.

[16] J. O. Thomas; R. Tellgren; I. Olovsson; (1974) CCDC 1592020: Hydrogen bond studies. LXXXIV. An X-ray diffraction study of the structures of KHCO3 and KDCO3 at 298, 219 and 95 K; Cambridge Crystallographic Data Centre. DOI: 10.1107/S0567740874004481.

[17] J. M. S. Skakle; M. Wilson; J. Feldmann; (2001). CCDC 2273323: Dipotassium carbonate sesquihydrate: rerefinement against new intensity data; Cambridge Crystallographic Data Centre. https://doi.org/10.1107/S1600536801016312.

[18] R. E. Dinnebier; S. Vensky; M. Jansen; P. Stephens; (2002). CCDC 1727671: Crystal structure of $K_2(C_2O_6)$ - first proof of existence and constitution of a peroxodicarbonate ion; Cambridge Crystallographic Data Centre. https://doi.org/10.1002/1521-3773(20020603)41:11<1922::AID-ANIE1922>3.0.CO;2-T.

[19] H. Ruette; D. Mootz; (1991). CCDC 1608305: Hydrate schwacher und starker Basen. V. Die Kristallstrukturen von KOH · $2H_2O$ (Substruktur) und KOH · $4H_2O$; Cambridge Crystallographic Data Centre. DOI: 10.1002/zaac.19916010109.

[20] F. Fillaux; A. Cousson; M. J. Gutman; (2008). CCDC 1676988: A neutron diffraction study of macroscopically entangled proton states in the high temperature phase of the KHCO3 crystal at 340 K. Cambridge Crystallographic Data Centre. DOI 10.1088/0953-8984/20/01/015225.

[21] F. Hinrichs; A. Adam; (2011). CCDC 1733732: A New Salt of the Monoperoxocarbonic Acid: $K_2(O_2)CO_2\cdot3.5H_2O_2$. Cambridge Crystallographic Data Centre. DOI: 10.1002/zaac.201000322.

[22] R.E.Dinnebier; S.Vensky; M.Jansen; J.C.Hanson; (2005). CCDC 1728885: Crystal Structures and Topological Aspects of the High-Temperature Phases and Decomposition Products of the Alkali-Metal Oxalates M2[C2O4] (M=K, Rb, Cs). Cambridge Crystallographic Data Centre. DOI: 10.1002/chem.200400616.

[23] H. Y. Becht, R. Struikmans, " A monoclinic high-temperature modification of potassium carbonate" *Acta Cryst. B* **1976**, *32*, 3344–3346.

[24] J. M. S. Skakle, M. Wilson, J. Feldman, " Dipotassium carbonate sesquihydrate: rerefinement against new intensity data" *Acta Cryst., E* **2001**, *57*, i94-i97.
